# Evaluation of diagnostic accuracy using routine laboratory parameters

**DOI:** 10.6026/973206300220683

**Published:** 2026-02-28

**Authors:** Trushna Shah, Anand Chauhan, Shyama Manojkumar Chag, Kirti Nishant Vyas

**Affiliations:** 1 Department of Biochemistry, Smt BK Shah Medical Institute and Research Center, Sumandeep Vidyapeeth Deemed to be University, Waghodia, Vadodara, Gujarat, India; 2 Department of Pathology, CU Shah Medical College, Surendranagar, Gujarat, India; 3 Department of Pathology, Pramukh Swami Medical College, Bhaikaka University, Karamsad, Gujarat, India; 4 Department of Pathology, Dr ND Desai Faculty of Medical Science and Research, Dharmsinh Desai University, Nadiad, Gujarat, India

**Keywords:** Diagnostic accuracy, laboratory parameters, early detection, screening, sensitivity, specificity

## Abstract

Early disease detection remains challenging in clinical practice, often leading to delayed diagnosis and poorer health outcomes. Therefore, it is of interest to evaluate the diagnostic accuracy of routine laboratory parameters for early disease detection among 486 adults attending a tertiary care hospital between January 2023 and December 2024. Standard laboratory investigations including complete blood count, metabolic panel, liver and kidney function tests and inflammatory markers were analyzed against confirmed diagnostic outcomes using sensitivity, specificity, predictive values and receiver operating characteristic analysis. Combined laboratory panels demonstrated significantly higher diagnostic accuracy (AUC = 0.847) than individual parameters, with inflammatory markers showing the highest sensitivity (82.4%) and the neutrophil-to-lymphocyte ratio providing the best sensitivity-specificity balance. Integration of multiple routine laboratory parameters may therefore offer a cost-effective and practical strategy for improving early disease detection in diverse healthcare settings.

## Background:

The timely identification of disease processes remains fundamental to effective clinical management and improved patient outcomes [1]. Routine laboratory investigations constitute an essential component of medical evaluation, providing objective data that guides diagnostic decision-making and therapeutic interventions [2]. Despite technological advances in molecular diagnostics and imaging modalities, basic laboratory parameters continue to serve as frontline screening tools in clinical practice due to their accessibility, cost-effectiveness and rapid turnaround time [3]. The global burden of chronic diseases has escalated dramatically, with cardiovascular disorders, malignancies, metabolic syndromes and infectious diseases accounting for significant proportions of morbidity and mortality [4]. Early detection during asymptomatic or minimally symptomatic phases substantially improves prognosis across disease categories. However, the diagnostic utility of routine laboratory parameters in identifying early-stage pathology has received variable attention in the literature [5]. Complete blood count analysis provides comprehensive hematological information that extends beyond detection of primary hematological disorders [6]. Derived indices such as the neutrophil-to-lymphocyte ratio and platelet-to-lymphocyte ratio have demonstrated predictive value for inflammatory conditions, malignancies and cardiovascular events [7]. Similarly, comprehensive metabolic panels encompassing hepatic and renal function markers offer insights into multi-organ dysfunction that may precede clinical manifestation [8].

Inflammatory biomarkers, including C-reactive protein and erythrocyte sedimentation rate, have established roles in disease monitoring but their utility in early detection screening remains incompletely characterized [9]. Recent investigations have explored integrated approaches combining multiple parameters to enhance diagnostic performance, recognizing that single-parameter analysis frequently lacks adequate sensitivity and specificity for reliable screening applications [10]. The concept of laboratory parameter panels for disease detection has evolved considerably, with mathematical modeling and machine learning approaches being applied to optimize parameter combinations [11]. Nevertheless, fundamental questions persist regarding the optimal configuration of routine tests for early disease identification across diverse clinical presentations [12]. Resource-constrained healthcare environments particularly benefit from evidence-based utilization of routine laboratory investigations, as advanced diagnostic technologies may be unavailable or economically unfeasible [13]. Establishing the diagnostic performance characteristics of commonly available tests supports rational test ordering and clinical interpretation [14]. A critical research gap exists in the systematic evaluation of routine laboratory parameters specifically for early disease detection across multiple disease categories simultaneously. Previous studies have predominantly focused on individual disease entities or specific parameter subsets, limiting generalizability and practical clinical application [15]. Therefore, it is of interest to evaluate and describe the diagnostic accuracy of routinely available laboratory parameters, individually and in combination, for early disease detection in a tertiary care clinical setting.

## Materials and Methods:

## Study design and setting:

This cross-sectional analytical study was conducted at the Department of Pathology and Internal Medicine, University Teaching Hospital, between January 2023 and December 2024. The institution serves as a referral center receiving patients from urban and rural catchment areas, ensuring demographic diversity within the study population.

## Sample size calculation:

Sample size was determined using the formula for diagnostic accuracy studies, considering an anticipated sensitivity of 75%, precision of 5% and 95% confidence interval. Accounting for potential incomplete data and loss to follow-up, a minimum sample of 450 participants was required. Recruitment continued until 486 eligible participants were enrolled.

## Participant selection:

## Inclusion criteria:

[1] Adults aged 18 years and above

[2] Presenting with non-specific symptoms warranting laboratory investigation

[3] Willingness to complete follow-up evaluation

[4] Absence of established chronic disease diagnosis at enrollment

## Exclusion criteria:

[1] Known pre-existing chronic medical conditions

[2] Pregnancy or lactation

[3] Acute trauma or surgical emergency

[4] Recent blood transfusion (within 3 months)

[5] Current immunosuppressive therapy

[6] Incomplete laboratory data

## Laboratory parameters evaluated:

Blood samples were collected following 8-12 hours fasting using standard phlebotomy protocols. Specimens were processed within 2 hours of collection according to laboratory standard operating procedures.

## Complete blood count panel:

[1] Hemoglobin, hematocrit, red blood cell count

[2] White blood cell count with differential

[3] Platelet count

[4] Red cell indices (MCV, MCH, MCHC, RDW)

[5] Derived ratios (NLR, PLR, MLR)

## Metabolic panel:

[1] Fasting blood glucose

[2] Glycated hemoglobin

[3] Lipid profile (total cholesterol, triglycerides, HDL, LDL)

[4] Serum electrolytes (sodium, potassium, chloride)

## Liver function tests:

[1] Serum bilirubin (total and direct)

[2] Alanine aminotransferase

[3] Aspartate aminotransferase

[4] Alkaline phosphatase

[5] Gamma-glutamyl transferase

[6] Total protein and albumin

## Kidney function tests:

[1] Serum creatinine

[2] Blood urea nitrogen

[3] Estimated glomerular filtration rate

## Inflammatory markers:

[1] C-reactive protein

[2] Erythrocyte sedimentation rate

[3] Ferritin

## Quality assurance:

All laboratory analyses were performed using calibrated automated analysers with internal and external quality control participation. Coefficient of variation for all parameters remained within acceptable limits throughout the study period.

## Diagnostic confirmation:

Participants underwent comprehensive clinical evaluation and additional investigations as clinically indicated. Final diagnostic outcomes were established through clinical assessment, imaging studies, histopathological examination and specialized testing according to disease category. Cases were classified as: confirmed disease, probable disease, or no significant pathology.

## Follow-up protocol:

Participants without immediate diagnosis underwent scheduled follow-up at 3, 6 and 12 months to capture delayed disease manifestation. Follow-up completion rate was 94.2%.

## Statistical analysis:

Data were analyzed using SPSS version 26.0 and MedCalc version 20.0. Continuous variables were expressed as mean±standard deviation or median with interquartile range based on distribution normality assessed by Shapiro-Wilk test. Categorical variables were presented as frequencies and percentages. Diagnostic accuracy metrics (sensitivity, specificity, positive predictive value, negative predictive value and diagnostic accuracy) were calculated for individual parameters and combined panels. Receiver operating characteristic curves were constructed with area under the curve quantification. Optimal cut-off values were determined using Youden's index. Chi-square test and independent t-test were applied for group comparisons. Multivariate logistic regression identified independent predictors of disease presence. Statistical significance was set at p < 0.05.

## Results:

A total of 486 participants completed the study protocol, comprising 258 males (53.1%) and 228 females (46.9%). Mean age was 47.3 ±14.6 years, ranging from 18 to 79 years. Following comprehensive evaluation, 214 participants (44.0%) received confirmed disease diagnoses, 67 (13.8%) were classified as probable disease and 205 (42.2%) showed no significant pathology. Disease categories among confirmed cases included metabolic disorders (n=72, 33.6%), inflammatory conditions (n=48, 22.4%), cardiovascular disease (n=38, 17.8%), malignancies (n=31, 14.5%) and infectious diseases (n=25, 11.7%) (Table 1). Significant differences were observed between disease-present and disease-absent groups across multiple laboratory parameters. Hemoglobin levels were significantly lower in the disease group (12.4±2.1 g/dL versus 13.8±1.4 g/dL, p < 0.001). White blood cell counts demonstrated elevation in disease-present participants (8.9±3.2 x 10^9^/L versus 6.7±1.8 x 10^9^/L, p < 0.001). The neutrophil-to-lymphocyte ratio showed marked elevation in the disease group (3.8±2.4 versus 1.9±0.8, p < 0.001). C-reactive protein levels were significantly higher among diseased participants (28.4± 35.6 mg/L versus 4.2±3.8 mg/L, p < 0.001). Liver transaminases, renal function parameters and metabolic markers also demonstrated statistically significant inter-group differences (Table 2). Receiver operating characteristic analysis revealed variable diagnostic performance across parameters. C-reactive protein demonstrated the highest area under the curve among individual parameters (AUC = 0.824, 95% CI: 0.786-0.862). The neutrophil-to-lymphocyte ratio achieved AUC of 0.792 (95% CI: 0.751-0.833), with optimal cut-off value of 2.5 yielding sensitivity of 76.2% and specificity of 72.4%. Fasting blood glucose showed AUC of 0.728 (95% CI: 0.682-0.774), while hemoglobin demonstrated AUC of 0.718 (95% CI: 0.671-0.765). Combined inflammatory panel (CRP + ESR + NLR) achieved the highest diagnostic accuracy with AUC of 0.847 (95% CI: 0.813-0.881) (Table 3). Logistic regression analysis identified independent predictors of disease presence. Elevated C-reactive protein (OR: 3.42, 95% CI: 2.18-5.36, p < 0.001), increased neutrophil-to-lymphocyte ratio (OR: 2.86, 95% CI: 1.84-4.44, p < 0.001), reduced hemoglobin (OR: 2.24, 95% CI: 1.48-3.39, p < 0.001) and elevated HbA1c (OR: 2.12, 95% CI: 1.42-3.16, p = 0.002) remained significant after adjustment for demographic variables. Diagnostic performance varied across disease categories. Inflammatory markers demonstrated highest sensitivity for inflammatory conditions (sensitivity 89.6%) and malignancies (sensitivity 84.5%). Metabolic parameters showed superior performance for metabolic disorders (AUC = 0.842). The combined panel maintained consistent accuracy across all disease categories.

## Discussion:

This comprehensive evaluation of routine laboratory parameters for early disease detection demonstrates the substantial diagnostic utility of commonly available tests when appropriately interpreted and combined. The findings support integration of multiple parameters into screening algorithms to optimize early identification of pathological processes across diverse disease categories. The superior diagnostic performance of inflammatory markers, particularly C-reactive protein and the neutrophil-to-lymphocyte ratio, aligns with contemporary understanding of inflammation as a common pathophysiological pathway across disease entities [16]. Systemic inflammatory responses accompany malignant transformation, metabolic dysregulation and cardiovascular pathology, rendering inflammatory indices valuable early detection tools [17]. The neutrophil-to-lymphocyte ratio demonstrated particular promise as a screening parameter, achieving favourable sensitivity-specificity balance with an optimal cut-off of 2.5. This finding corresponds with accumulating evidence supporting NLR utility in prognostic stratification and disease prediction [18]. The accessibility and cost-effectiveness of this derived parameter enhances its practical applicability, especially in resource-limited settings where advanced biomarkers remain unavailable [19]. Combined laboratory panels achieved substantially higher diagnostic accuracy compared to individual parameters, confirming the value of integrated interpretation. The synergistic effect of multiple parameters compensates for individual test limitations, improving overall screening performance [20]. Modern computational approaches, including machine learning algorithms, may further optimize parameter combinations for specific clinical applications [21]. Metabolic parameters demonstrated significant associations with disease presence, reflecting the interconnection between metabolic dysfunction and multiple pathological processes. Glycemic markers and lipid profiles contribute not only to cardiovascular risk assessment but also to broader disease susceptibility evaluation [22]. The observed elevation of fasting glucose and HbA1c among diseased participants underscores the importance of metabolic screening in general health assessment [23]. Haematological parameters, including hemoglobin concentration and red cell distribution width, provided valuable diagnostic information. Anemia frequently accompanies chronic disease states and early detection of hemoglobin decline may prompt investigation of underlying pathology [24]. The red cell distribution width has emerged as a marker of systemic inflammation and oxidative stress, extending its utility beyond traditional hematological applications [25]. Hepatic and renal function parameters demonstrated moderate diagnostic accuracy individually but contributed meaningfully to combined panel performance. Subclinical organ dysfunction may precede overt disease manifestation and laboratory detection enables early intervention [26].

The incorporation of estimated glomerular filtration rate and transaminase levels into screening protocols supports comprehensive health evaluation [27]. Age-related differences in diagnostic performance were observed, with younger participants showing higher specificity and older participants demonstrating higher sensitivity. This observation necessitates age-adjusted interpretation of laboratory results in screening contexts [28]. Development of age-specific reference ranges and cut-off values may optimize diagnostic accuracy across age strata [29]. The practical implications of these findings extend to clinical guideline development and healthcare resource allocation. Evidence-based laboratory utilization reduces unnecessary testing while ensuring appropriate investigation of at-risk individuals [30]. Implementation of standardized screening panels based on demonstrated diagnostic performance supports efficient clinical practice [31]. Study limitations include the single-center design potentially limiting generalizability, cross-sectional assessment precluding evaluation of temporal relationships and heterogeneous disease categories complicating unified analysis. Additionally, the 12-month follow-up period may have been insufficient to capture slowly progressive pathology. Future multicenter prospective investigations with extended follow-up are warranted. The evolving integration of artificial intelligence and machine learning in laboratory medicine offers opportunities to enhance diagnostic accuracy through sophisticated pattern recognition [32]. Algorithm development incorporating routine laboratory parameters with clinical and demographic variables may achieve diagnostic performance approaching advanced biomarker panels [33].

## Conclusion:

We show that combined interpretation of routine laboratory parameters provides meaningful diagnostic accuracy for early disease detection in clinical practice. Inflammatory markers and hematological indices, particularly the neutrophil-to-lymphocyte ratio and C-reactive protein, showed strong screening utility when used as part of integrated laboratory panels. Adoption of structured, panel- based laboratory assessment may enhance early diagnosis in a cost-effective manner, especially in resource-limited healthcare settings.

## Figures and Tables

**Table 1:** Demographic and baseline clinical characteristics of study participants

**Variable**	**Total (n=486)**	**Disease Present (n=214)**	**Disease Absent (n=205)**	**p-value**
Age (years), mean±SD	47.3±14.6	52.8±13.2	41.5±14.1	<0.001
Gender (Male), n (%)	258 (53.1)	124 (57.9)	98 (47.8)	0.038
BMI (kg/m^2^), mean±SD	26.4±4.8	28.1±5.2	24.6±3.9	<0.001
Systolic BP (mmHg)	128.5±18.3	135.6±19.4	121.2±14.8	<0.001
Diastolic BP (mmHg)	82.4±11.2	86.3±12.1	78.4±9.2	<0.001
Smoking history, n (%)	112 (23.0)	68 (31.8)	32 (15.6)	<0.001
Family history, n (%)	156 (32.1)	89 (41.6)	48 (23.4)	<0.001

**Table 2:** Comparison of laboratory parameters between groups

**Parameter**	**Disease Present (n=214)**	**Disease Absent (n=205)**	**p-value**
Hemoglobin (g/dL)	12.4±2.1	13.8±1.4	<0.001
WBC (x10^9^/L)	8.9±3.2	6.7±1.8	<0.001
Platelet count (x10^9^/L)	268±98	242±62	0.002
NLR	3.8±2.4	1.9±0.8	<0.001
PLR	168±78	124±42	<0.001
FBG (mg/dL)	118.6±42.3	94.2±12.8	<0.001
HbA1c (%)	6.4±1.8	5.4±0.5	<0.001
Total cholesterol (mg/dL)	218±48	186±32	<0.001
ALT (U/L)	42.8±28.4	24.6±12.2	<0.001
AST (U/L)	38.4±24.6	22.8±8.6	<0.001
Creatinine (mg/dL)	1.14±0.48	0.88±0.18	<0.001
eGFR (mL/min/1.73m^2^)	78.4±24.6	96.2±15.8	<0.001
CRP (mg/L)	28.4±35.6	4.2±3.8	<0.001
ESR (mm/hr)	34.8±26.4	12.4±8.6	<0.001

**Table 3:** Diagnostic performance characteristics of laboratory parameters

**Parameter**	**Optimal Cut-off**	**Sensitivity (%)**	**Specificity (%)**	**PPV (%)**	**NPV (%)**	**AUC (95% CI)**
Hemoglobin	<12.5 g/dL	68.2	74.6	73.8	69.2	0.718 (0.671-0.765)
WBC	>8.0 x10^9^/L	62.4	78.2	74.8	66.8	0.742 (0.698-0.786)
NLR	>2.5	76.2	72.4	74.2	74.5	0.792 (0.751-0.833)
PLR	>145	64.8	71.6	70.4	66.2	0.712 (0.664-0.760)
FBG	>105 mg/dL	58.4	86.8	82.2	66.4	0.728 (0.682-0.774)
HbA1c	>5.9%	62.6	84.4	80.8	68.4	0.764 (0.720-0.808)
ALT	>35 U/L	56.8	82.4	77.2	64.6	0.698 (0.649-0.747)
Creatinine	>1.1 mg/dL	48.6	88.2	81.2	62.1	0.684 (0.634-0.734)
CRP	>8 mg/L	82.4	76.8	78.8	80.8	0.824 (0.786-0.862)
ESR	>20 mm/hr	72.6	74.2	74.6	72.2	0.768 (0.724-0.812)
Combined Panel	-	86.4	78.6	80.8	84.8	0.847 (0.813-0.881)
